# Sustained resveratrol infusion increases natriuresis independent of renal vasodilation

**DOI:** 10.14814/phy2.12144

**Published:** 2014-09-11

**Authors:** Kevin L. Gordish, William H. Beierwaltes

**Affiliations:** 1 Department of Physiology, Wayne State University School of Medicine, Detroit, Michigan; 2 Department Internal Medicine, Hypertension and Vascular Research Div., Henry Ford Hospital, Detroit, Michigan

**Keywords:** Glomerular filtration, natriuresis, nitric oxide, renal blood flow

## Abstract

Resveratrol is reported to exert cardio‐renal protective effects in animal models of pathology, yet the mechanisms underlying these effects are poorly understood. Previously, we reported an *i.v*. bolus of resveratrol induces renal vasodilation by increasing nitric oxide bioavailability and inhibiting reactive oxygen species. Thus, we hypothesized a sustained infusion of resveratrol would also increase renal blood flow (RBF), and additionally glomerular filtration rate (GFR). We infused vehicle for 30 min followed by 30 min resveratrol at either: 0, 0.5, 1.0, 1.5 mg/min, and measured RBF, renal vascular resistance (RVR), GFR, and urinary sodium excretion. At all three doses, blood pressure and GFR remained unchanged. Control RBF was 7.69 ± 0.84 mL/min/gkw and remained unchanged by 0.5 mg/min resveratrol (7.88 ± 0.94 mL/min/gkw, *n *= 9), but urinary sodium excretion increased from 2.19 ± 1.1 to 5.07 ± 0.92 *μ*mol/min/gkw (*n *= 7, *P *< 0.01). In separate experiments, 1.0 mg/min resveratrol increased RBF by 17%, from 7.16 ± 0.29 to 8.35 ± 0.42 mL/min/gkw (*P *< 0.01, *n *= 10), decreased RVR 16% from 13.63 ± 0.65 to 11.36 ± 0.75 ARU (*P *< 0.003) and increased sodium excretion from 1.57 ± 0.46 to 3.10 ± 0.80 *μ*mol/min/gkw (*n *= 7, *P *< 0.04). At the 1.5 mg/min dose, resveratrol increased RBF 12% from 6.76 ± 0.57 to 7.58 ± 0.60 mL/min/gkw (*n *= 8, *P* < 0.003), decreased RVR 15% (15.58 ± 1.35 to 13.27 ± 1.14 ARU,* P* < 0.003) and increased sodium excretion (3.99 ± 1.71 to 7.80 ± 1.51 *μ*mol/min/gkw, *n *= 8, *P* < 0.04). We conclude that a constant infusion of resveratrol can induce significant renal vasodilation while not altering GFR or blood pressure. Also, resveratrol infusion produced significant natriuresis at all doses, suggesting it may have a direct effect on renal tubular sodium handling independent of renal perfusion pressure or flow.

## Abstract (precis)

We have previously documented that resveratrol causes a nitric oxide‐dependent acute renal vasodilation. We now report that sustained resveratrol has no effect on GFR but induced a remarkable natriuresis which is independent from the hemodynamic effects, suggesting resveratrol acts directly on nephron sodium reabsorption.

## Introduction

Resveratrol (3,5,4′‐trihydroxystilbene), a polyphenol found in red wine and other foods, is often attributed to providing cardioprotective effects through nitric oxide‐mediated vascular relaxation and possible antioxidant properties (Baur and Sinclair 2006). However, little is known about its effects on the kidney. Recently, we reported an i.v. bolus of resveratrol induces renal vasodilation by increasing nitric oxide (NO) availability and inhibiting generation of reactive oxygen species (Gordish and Beierwaltes 2014).

NO has an important role in the control of renal function and long‐term regulation of blood pressure (Persson 2002). NO has multiple effects within the kidney including increasing renal blood flow (RBF) (Navar et al. 1996), increasing glomerular filtration rate (GFR) (Gabbai and Blantz 1999), and inhibiting thick ascending limb (TAL) sodium reabsorption (Herrera et al. 2006). NO, a vasodilator, is tonically produced in the vascular endothelium by the enzyme endothelial nitric oxide synthase (eNOS) and synthesized from the amino acid substrate L‐arginine (Palmer et al. 1988). Inhibition of NOS results in acute hypertension and renal vasoconstriction (Baylis et al. 1990; Beierwaltes et al. 1992). Thus, the renal vascular endothelium maintains a tonic balance between vasodilatation and vasoconstriction.

The existing literature supports an interactive involvement between resveratrol and nitric oxide. Resveratrol increases eNOS expression and when incubated with human umbilical vein endothelial cells can acutely increase NO synthesis (Wallerath et al. 2002, 2003). NO production is well‐established as an endothelial‐dependent vasodilator and *N*‐nitro‐L‐arginine methyl ester hydrochloride (L‐NAME), a NOS inhibitor, reverses this effect (Chen and Pace‐Asciak 1996). At a molecular level, nanomolar concentrations of resveratrol phosphorylates eNOS at serine 1177, thereby increasing eNOS activity (Dolinsky et al. 2013). Consistent with our data in the kidney (Gordish and Beierwaltes 2014), these data suggest resveratrol vasodilates through NO dependent mechanisms.

Relatively few studies have investigated the effects of resveratrol on renal hemodynamics and glomerular filtration, especially in normal rat. NO synthesis within the kidney contributes toward basal tone, enhancing RBF, and GFR (Lahera et al. 1991). In a rat model of acute gentamicin‐induced kidney failure, 5 days of resveratrol treatment improved diminished RBF and improved GFR (Morales et al. 2002). Similarly, in a rat model of sepsis‐induced acute kidney injury, resveratrol treatment increased RBF and partially restored diminished GFR (Holthoff et al. 2012). Also, resveratrol marginally improved glomerular filtration in an acute model of renal failure induced by cisplatin (Kim et al. 2011). Combined, these data suggest resveratrol, in various animal models of compromised renal function, may increase RBF and partially restore GFR after an insult. However, the effects of resveratrol on GFR in normal (uncompromised) rat kidneys remain unclear.

NO also plays a critical role in salt and water transport along the nephron (Ortiz and Garvin 2002). NO enhances natriuresis through inhibition of sodium reabsorption along segments of the nephron (Mattson et al. 1992). eNOS generated NO inhibits sodium reabsorption in the thick ascending limb (TAL) (Ortiz et al. 2003). However, it is not known if resveratrol influences NO synthesis in these sites.

On the basis of these observations, we hypothesized a sustained resveratrol infusion would increase RBF and also GFR. Furthermore, we hypothesized resveratrol, independent of its hemodynamic effects, may increase sodium excretion.

## General Methods

All protocols and surgical procedures employed in this study were reviewed and approved by the Henry Ford Health System Institutional Animal Care and Use Committee (IACUC) and were performed in accordance with the *Guide for the Care and Use of Laboratory Animals* endorsed by the American Physiological Society in accordance with National Institutes of Health guidelines.

Male Sprague Dawley rats (Charles River, Wilmington, MA) of 300–400 g body weight (b.w.) were fasted overnight, but allowed free access to drinking water. On the day of the experiment, rats were anesthetized *via* intraperitoneal injection with thiobutabarbital, 125 mg/kg b.w. (Inactin, Sigma Aldrich, St. Louis, MO). Rats were placed on a heated surgical table to maintain constant body temperature (BrainTree Scientific, Braintree, MA). A tracheotomy was performed using PE‐240 tubing to allow free breathing of room air. A femoral cut down was performed to cannulate the femoral artery and vein with PE‐50 catheters (Becton Dickinson, Franklin Lakes, NJ). The arterial catheter was connected to an iWorx BP‐102 probe with LabScribe2 software (iWorx, Dover, NH) for simultaneous recording of mean arterial pressure (MAP) and also used for blood collection. Pressure transducers were calibrated using a digital, mercury‐free Traceable manometer (Fisher Scientific, Pittsburg, PA). The femoral venous catheter was used for a 1 mL postsurgical supplement of 6% bovine serum albumin (BSA) (Sigma Aldrich) and for constant infusion of either vehicle or resveratrol plus inulin. A midventral abdominal incision was performed and the intestines wrapped in moist gauze and moved to the right side of the peritoneal cavity to expose the left kidney. The left renal artery and vein were carefully isolated and the renal artery was fitted with a Doppler flow probe (Transonic, Ithaca, NY) connected to a transit‐time perivascular flow meter TS‐420 model (Transonic) and the iWorx data acquisition system to record renal blood flow (RBF). The bladder was exposed through a suprapubic incision and was cannulated with a 23‐gauge needle connected to PE‐50 tubing. It was secured with Vetbond (3M, St. Paul, MN). The rat was draped and allowed to stabilize for 20–30 min prior to running the experimental protocols.

### Protocol 1: Renal hemodynamics with resveratrol

We hypothesized sustained resveratrol would increase RBF and GFR. To test the effects of resveratrol on renal hemodynamics, we employed three different doses (0.5, 1.0, and 1.5 mg/min) using three separate groups of rats; one for each dose (*n *= 7, 10, and 7, respectively). Following surgical stabilization, we intravenously infused vehicle at a rate of 80 *μ*l/min for 30 min followed by a 30 min resveratrol infusion. Recordings for the resveratrol infusion period began after readings stabilized between the changing of the infusion syringes. Data presented for vehicle or resveratrol periods are 30 min averages. FITC‐inulin was infused throughout the protocols. RBF and mean arterial pressure (MAP) were recorded. Renal vascular resistance (RVR) was calculated by dividing MAP by RBF in units of mmHg·mL/min·gram of kidney/weight (gkw), hereafter referred to as arbitrary resistance units (ARU).

GFR was measured by the clearance of FITC‐inulin (Sigmon and Beierwaltes 2000). Arterial blood was sampled and urine was collected during both the vehicle and resveratrol infusions. Plasma and urine inulin fluorescence was assessed with a Synergy H1 Microplate Reader (BioTek, Winooski, VT) to calculate plasma and urine inulin concentrations.

Urinary sodium concentrations were measured with a Nova1 autoanalyzer (Nova Biomedical, Waltham, MA). Urinary sodium excretion was calculated from urine volume, collection time, and urine sodium concentration.

At the conclusion of the protocol, animals were terminated by barbiturate overdose and aortic transection. The kidneys were removed, decapsulated, blotted, and weighed.

The resveratrol (Cayman Chemical, Ann Arbor, MI) solution was prepared daily. Resveratrol was dissolved in 4 mL of DMSO and diluted with 5.6 mL of 0.9% saline (final concentration of DMSO was approximately 42%). The resveratrol solution was wrapped in aluminum foil and kept at 37°C until it was used. Vehicle periods used the saline plus DMSO cocktail without resveratrol.

### Protocol 2: Renal hemodynamics with vehicle

We performed a set of vehicle infusion controls to serve as controls for protocol 1. Vehicle, as in protocol 1, contained 4 mL of DMSO and 5.6 mL 0.9% saline. We infused vehicle at the same rate of 80 *μ*L/min plus inulin, but vehicle infusion was maintained throughout the entire protocol (without the addition of resveratrol as above). As above, the time course for vehicle controls was similar to the experimental protocol. Measurements were similar to those in protocol 1.

### Analysis

The resveratrol response was compared to matched vehicle periods using a paired Student's *t*‐test with an *α* acceptance at 0.05, and *n* values were chosen to provide a power of at least 0.8.

## Results

### Renal hemodynamics with resveratrol

**Figure 1.**
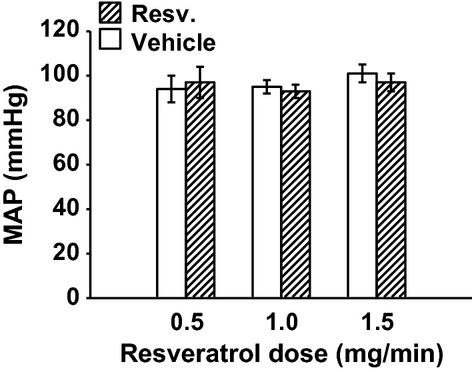
Mean arterial pressure (MAP) with intravenous vehicle (30 min) followed by resveratrol (Resv.) infusion (30 min) in three different groups (*n *= 7, 10, and 7, respectively) at three different doses. Resveratrol infusion had no effect on MAP.

**Figure 2.**
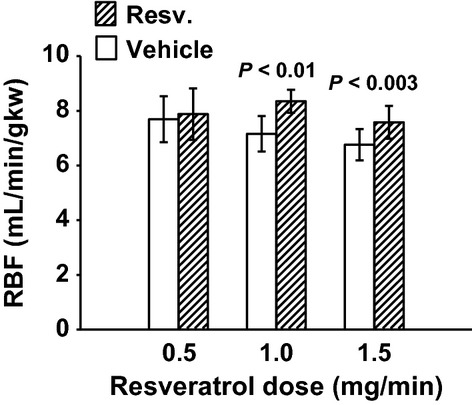
Renal blood flow (RBF) in response to vehicle (30 min) followed by resveratrol (Resv.) infusion (30 min). The lower dose of 0.5 mg/min had no effect on RBF, but both higher doses increased RBF by 17% and 12%, respectively.

**Figure 3.**
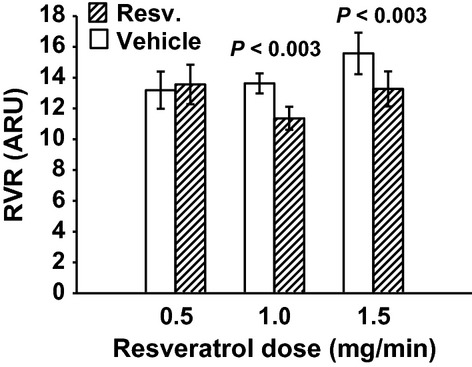
Renal vascular resistance (RVR) in response to vehicle (30 min) followed by resveratrol (Resv.) infusion (30 min). The lower dose of 0.5 mg/min had no effect on RVR, but both higher doses decreased RVR by 16% and 15%, respectively.

**Figure 4.**
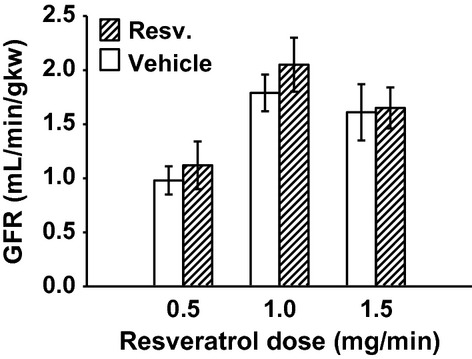
Glomerular Filtration Rate (GFR) during vehicle (30 min) followed by resveratrol (Resv.) infusion (30 min). Resveratrol infusion had no effect on GFR at any dose.

**Figure 5.**
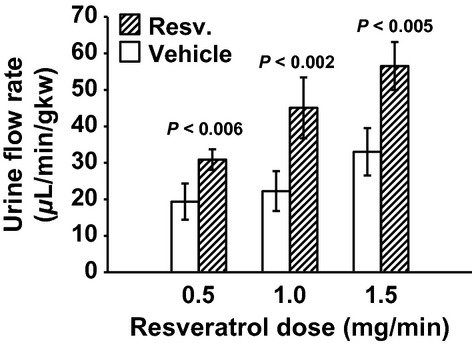
Urine Flow Rate in response to vehicle (30 min) followed by resveratrol (Resv.) infusion (30 min). Resveratrol infusion rate increased urine flow in all three groups by 59%, 103%, and 71%, respectively.

**Figure 6.**
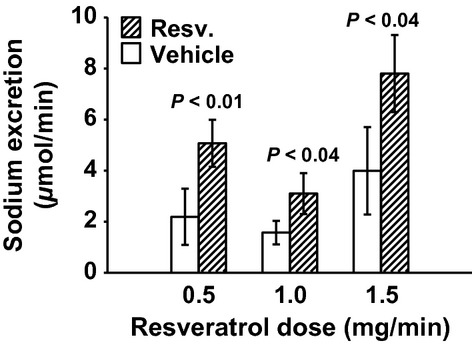
Urinary sodium excretion in response to vehicle (30 min) and resveratrol (Resv.) infusion (30 min). All three doses of resveratrol infusion increased sodium excretion, by 132%, 97%, and 95%, respectively.

MAP was unchanged with 0.5 mg/min resveratrol (Fig. 1). RBF was also not changed by 0.5 mg/min resveratrol (7.69 ± 0.84 vs. 7.88 ± 0.94 mL/min/gkw, *n *= 9) (Fig. 2), and thus RVR remained unchanged (Fig. 3). In this group, GFR was 0.98 ± 13 mL/min/gkw (Fig. 4) and was unchanged by the lower dose of resveratrol infusion. However, urine flow rate was increased by 60% (19.37 ± 4.97 to 30.89 ± 2.81 *μ*L/min/gkw, [Fig. 5] *P* < 0.006), and urinary sodium excretion increased by 2.3‐fold, from 2.19 ± 1.1 to 5.07 ± 0.92 *μ*mol/min/gkw (Fig. 6, *n *= 7, *P* < 0.01).

In separate experiments, 1.0 mg/min resveratrol had no effect on MAP (Fig. 1), but RBF increased by 17%, from 7.16 ± 0.29 to 8.35 ± 0.42 mL/min/gkw (Fig. 2, *P* < 0.01, *n *= 10) and RVR decreased 16% (Fig. 3) from 13.63 ± 0.65 to 11.36 ± 0.75 ARU (*P *< 0.003). However, GFR remained unchanged (Fig. 4, 1.79 ± 0.17 vs. 2.05 ± 0.25 mg/min/gkw). Urine flow rate doubled from 22.24 ± 5.47 to 45.07 ± 8.36 *μ*L/min/gkw (Fig. 5, *n *= 7, *P *< 0.002), and sodium excretion increased from 1.57 ± 0.46 to 3.10 ± 0.80 *μ*mol/min/gkw (Fig. 6, *n *= 7, *P *< 0.04).

In rats given the higher 1.5 mg/min dose of resveratrol, again there was no effect on MAP (Fig. 1). Similar to the mid‐dose, resveratrol increased RBF 12% from 6.76 ± 0.57 to 7.58 ± 0.60 mL/min/gkw (Fig. 2, *n *= 8, *P *< 0.003) and thus RVR decreased 15% (Fig. 3) from 15.58 ± 1.35 to 13.27 ± 1.14 ARU (*P *< 0.003). As before, GFR remained unchanged (Fig. 4, 1.61 ± 0.26 vs. 1.65 ± 0.19 mL/min/gkw). Urine flow rate increased 70% (Fig. 5) from 33.01 ± 6.48 to 56.53 ± 6.56 *μ*L/min/gkw (*n *= 8, *P *< 0.005) and sodium excretion nearly doubled (Fig. 6) from 3.99 ± 1.71 to 7.80 ± 1.51 *μ*mol/min/gkw (*n *= 8, *P *< 0.04).

### Protocol 2: Renal hemodynamics with vehicle

In vehicle time controls, (Table 1), MAP was unchanged and both RBF and RVR remained constant. With the vehicle infusion, urine flow rate increased 20% from 42.83 ± 5.36 to 53.08 ± 7.62 *μ*L/min/gkw (*P *< 0.04), but sodium excretion remained unchanged. Our vehicle including DMSO had no overall effect on MAP, RBF, or RVR.

**Table 1.** Time control renal hemodynamics and excretion with vehicle only.

	Vehicle period 1 (30 min)		Vehicle period 2 (30 min)
MAP (mL/min/gkw)	104 ± 8	N.S.	105 ± 8
RBF (mL/min/gkw)	7.25 ± 0.98	N.S.	7.02 ± 1.17
RVR (ARU)	14.76 ± 1.47	N.S.	15.79 ± 2.23
Urine flow rate (*μ*L/min/gkw)	42.83 ± 5.36	*P* < 0.04	53.08 ± 7.62
Sodium excretion (*μ*mol/min)	3.24 ± 1.10	N.S.	4.93 ± 1.62

## Discussion

Very little is known about the effects of resveratrol on renal function, especially in the normal state in the absence of renal pathology. We previously reported an i.v. bolus of resveratrol produced an acute renal vasodilation that was mediated by increased NO bioavailability and scavenging of reactive oxygen free radicals. This study expands upon these observations, and addresses resveratrol‐induced changes in renal function in response to sustained infusion. Notably, we still find significant renal vasodilation, but surprisingly without changes in GFR. Additionally, we found resveratrol produced a significant diuresis and natriuresis, and this can occur independent of hemodynamic changes.

Resveratrol has been reported to lower blood pressure, but these observations are primarily in animal models of hypertension (Thandapilly et al. 2012; Dolinsky et al. 2013; Cheng et al. 2014) or diabetes with hypertension as a comorbidity (Rivera et al. 2009). Translational studies with human participants are sparse and offer mixed results (Poulsen et al. 2013; Wong et al. 2013). Chronic resveratrol supplementation (75 mg/day) for 6 weeks in mildly hypertensive obese human subjects did not lower blood pressure (Wong et al. 2013) and another study using chronic resveratrol (500 mg 3×/day) for 4 weeks also did not reduce blood pressure in obese men with prehypertension (Poulsen et al. 2013). In our present acute protocol using different doses of sustained resveratrol infusion, our normotensive rats did not have any change in blood pressure.

In our previous work (Gordish and Beierwaltes 2014), we addressed responsible mechanisms for renal vasodilation. We found it was in part due to increased NO synthesis or availability and also involved a reduction in reactive oxygen species. In this study, we infused resveratrol hypothesizing it would increase RBF which would lead to increased GFR. Interactions between vasoconstriction and vasodilation (including nitric oxide) in the kidney plays a role in regulation of renal hemodynamics (Sigmon et al. 1992; Sigmon and Beierwaltes 1993; Baylis et al. 1994; Herrera and Garvin 2005). Our low dose of 0.5 mg/min resveratrol did not increase RBF or decrease RVR. However, the higher doses of resveratrol (1.0 and 1.5 mg/min) significantly increased RBF and decreased RVR, consistent with our previous findings (Gordish and Beierwaltes 2014). The present results show renal vasodilation in response to resveratrol is maintained during constant infusion at the mid and high dose. However, there appears to be a maximal limit to resveratrol‐induced renal vasodilation in a normotensive rat, as infusion at the mid and high dose both reduced renal vascular resistance by 16% and 15%, respectively; similar to the changes seen in response to an acute bolus (Gordish and Beierwaltes 2014). Resveratrol‐induced increases in perfusion may be advantageous in compromised renal states when there is lower nitric oxide levels and increased oxidative stress as is often the case in hypertension (Hall 1986) and other renal diseases (Forbes et al. 2008).

Nonselective NO inhibition is found to increase RVR and decrease glomerular filtration rate (Bech et al. 1996; Gabbai and Blantz 1999). We hypothesized that resveratrol‐induced increases in RBF (due to increased NO) would lead to increased GFR, but this was not supported by our data, as all three resveratrol doses failed to increase GFR. It is not clear why the increased RBF is uncoupled from GFR in these studies. A possible explanation may lay in that while resveratrol reduced afferent arteriolar resistance and increased RBF, the efferent arterioles (in the normal rat) may have also dilated to maintain glomerular filtration at a constant rate. Thus, while resveratrol may rescue GFR in models of compromised renal function (Morales et al. 2002; Kim et al. 2011; Holthoff et al. 2012), it had no effect in a normal, intact kidney.

The most novel finding of these studies was the significant resveratrol‐induced diuresis and natriuresis, even in the absence of hemodynamics changes. This increase in urine flow rate and urinary sodium excretion is also likely due to the effects of renal NO. An extensive review by Li and Förstermann (2009) on resveratrol and endothelial function details numerous pathways in which resveratrol increases NO synthesis and bioavailability. Perez‐Rojas et al. (2010) demonstrated (using NOS‐3 knock‐out mice) how NO within the kidney promotes water and sodium excretion. Resveratrol may possibly be acting as a natriuretic by stimulating NO production in the nephron as it does in the endothelium (Gordish and Beierwaltes 2014).

NO is important regulator of thick ascending limb (TAL) sodium transport where approximately 20–30% of filtered sodium is reabsorbed (Greger and Velazquez 1987). NO has been shown to inhibit sodium reabsorption within the TAL (Ortiz and Garvin 2001). Majid et al. (1993) have shown when NO is inhibited in dogs, renal blood flow, urine flow, and sodium excretion are decreased, suggesting NO has both diuretic and natriuretic effects in vivo. Thus, NO changes within the TAL are physiologically relevant. Increased shear stress in primary culture of TAL cells has been shown to stimulate the production of NO by NOS 3 (Cabral et al. 2010) and increased renal luminal flow also stimulates NO production within in the TAL (Cabral et al. 2012). In our previous work (Gordish and Beierwaltes 2014), we demonstrated resveratrol increased renal vasodilation due to increased NO synthesis or availability and through reductions in reactive oxygen species including the possibility of superoxide formation. Changes in tubular flow‐induced stretch and sodium chloride delivery stimulate superoxide production by nicotinamide adenine dinucleotide phosphate (NADPH) oxidase in the TAL. Three isoforms of NADPH oxidase are present within the TAL (Massey et al. 2012). When NADPH oxidase 4 (Nox4) was silenced superoxide production was decreased, suggesting Nox4 mediates flow‐induced superoxide production in the TAL (Hong and Garvin 2012). Resveratrol, in addition to increasing NO, has been shown to decrease NOX activity (Chow et al. 2007) and NOX4 expression (Spanier et al. 2009). Our whole animal studies do not allow us to pin‐point the tubular site of action of the resveratrol‐induced natriuresis, but our results are consistent with the literature suggesting NO acts within the TAL (Hong and Garvin 2012; Massey et al. 2012). It remains unclear if the diuretic and natriuretic effects of resveratrol are due to increased NO availability, reduced superoxide production, or a combination of the above mechanisms as seen in the renal vasculature (Gordish and Beierwaltes 2014). Overall, the data presented in our study are consistent with resveratrol‐induced tubular NO synthesis promoting diuresis and natriuresis.

In our study, the resveratrol‐induced natriuretic effect was observed with all three resveratrol doses. The increases in urinary sodium excretion were dissociated from changes in blood flow. Separate from the effects of NO, resveratrol may affect renal tubular sodium handling through direct inhibition of sodium transport. Weixel et al. (2013) has shown that resveratrol inhibits Epithelial Sodium Channel (ENaC) in mouse cortical collecting duct cells. It remains unknown if ENaC is being inhibited in vivo. However, this may explain how resveratrol may contribute to lowering blood pressure in animal models of hypertension, particularly when salt‐sensitive hypertension is present (Miatello et al. 2005; Rivera et al. 2009; Dolinsky et al. 2013; Leibowitz et al. 2014).

In summary our findings suggest a constant infusion of resveratrol induced significant renal vasodilation while not altering either GFR or blood pressure in normal rats. Additionally, resveratrol infusion produced significant natriuresis at all doses, independent of hemodynamic responses, suggesting it may have a direct effect on renal tubular sodium handling independent of perfusion pressure, RBF, or changes in RVR.

## Conflicts of Interest

None declared.
